# Association of Autoantibodies against M2-Muscarinic Acetylcholine Receptor with Atrial Fibrosis in Atrial Fibrillation Patients

**DOI:** 10.1155/2019/8271871

**Published:** 2019-02-04

**Authors:** Guiling Ma, Xuejiao Wu, Lijun Zeng, Jiawei Jin, Xingpeng Liu, Jianjun Zhang, Lin Zhang

**Affiliations:** ^1^Heart Center, Beijing Chao-Yang Hospital, Capital Medical University, Beijing 100043, China; ^2^Institute for Medical Research, Beijing Chao-Yang Hospital, Capital Medical University, Beijing 100043, China

## Abstract

**Objectives:**

To investigate the association of serum autoantibodies against M2-muscarinic acetylcholine receptor (anti-M2-R) with atrial fibrosis in long-standing persistent atrial fibrillation (AF) patients.

**Methods:**

Twenty-four long-standing persistent AF patients, scheduled to undergo hybrid ablation surgery, were enrolled in the study. Twenty-six patients with sinus rhythm, scheduled to undergo coronary artery bypass grafting surgery, were enrolled into the non-AF group. We detected serum anti-M2-R levels. Left atrial appendages were subjected to histological and molecular biological assays. Patients in the AF group received follow-up for two years.

**Results:**

The AF group showed significantly higher serum anti-M2-R levels compared to the non-AF group (496.2 ± 232.5 vs. 86.3 ± 25.7 pmol/L, *p* < 0.001). The AF group exhibited severe fibrosis in the left atrial appendages, as indicated by increased collagen volume fraction (45.2 ± 4.7% vs. 27.6 ± 8.3%, *p* < 0.001), and higher levels of collagen I (0.52 ± 0.04 vs. 0.24 ± 0.06, *p* < 0.001) and collagen III (0.51 ± 0.07 vs. 0.36 ± 0.09, *p* < 0.001). TGF-*β*1 and CTGF were also upregulated in the AF group. A positive correlation between serum anti-M2-R levels and fibrosis of the left atrial appendage and fibrogenic indexes was observed.

**Conclusions:**

Serum anti-M2-R levels are higher in AF patients and are associated with the severity of atrial fibrosis. In addition, serum anti-M2-R levels are positively correlated to TGF-*β*1 and CTGF expression in the left atrial appendage.

## 1. Introduction

Atrial fibrillation (AF) is the most common arrhythmia in the clinical setting. The estimated number of AF patients worldwide in 2010 was 33.5 million, which included 20.9 million males and 12.6 million females [1]. The incidence and prevalence rates of AF are generally higher in developed countries. Approximately 25% of middle-aged adults in Europe and the USA are predicted to develop AF, with higher prevalence in older people and in patients with hypertension, diabetes mellitus, coronary artery disease, or heart failure [2–4]. The underlying pathogenesis of AF has long been disputed, but atrial remodeling, including electrical, structural, and autonomic remodeling, has always been recognized to contribute to the maintenance of AF in animal models and humans [5]. There is growing evidence confirming that autoimmunity may play an important role in the initiation and perpetuation of AF [6].

M2-muscarinic acetylcholine receptor belongs to the family of cardiac G-protein-coupled receptors. Circulating autoantibodies against M2-muscarinic acetylcholine receptors (anti-M2-R) have been found in several cardiac arrhythmias [7]. Our previous study showed that serum anti-M2-R levels are higher in AF patients and positive for anti-M2-R as an independent predictor for the recurrence of AF one year after radiofrequency catheter ablation [8]. A recent report has shown that serum anti-M2-R has a predictive value for moderate-extensive left atrial fibrosis defined by delayed-enhancement magnetic resonance imaging (DE-MRI) in patients following cryoablation [9]. Atrial fibrosis is one of the most fundamental mechanisms involved in the physiopathology of AF [10]. TGF-*β*1 and connective tissue growth factor (CTGF) are strong fibrogenic indexes. Studies have shown that plasma TGF-*β*1 and CTGF levels are correlated with the degree of left atrial fibrosis and are associated with the development and maintenance of AF [11–13]. However, the relationship between anti-M2-R and atrial fibrosis and the association between anti-M2-R and fibrogenic indexes, such as TGF-*β*1 and CTGF, in AF patients are not clear.

Therefore, the aim of this study was to test the hypothesis that anti-M2-R is associated with atrial fibrosis. We tested serum anti-M2-R levels of AF patients, non-AF patients, and healthy controls by ELISA. We assessed atrial fibrosis and examined the expression of M2 receptor, TGF-*β*1, CTGF, collagen I, and collagen III in the left atrial appendage (LAA) of long-standing persistent AF patients receiving hybrid ablation surgery and patients receiving coronary artery bypass grafting (CABG) surgery with sinus rhythm. The relationship between anti-M2-R and fibrogenic indexes was also investigated.

## 2. Materials and Methods

### 2.1. Study Population

During the period between January 2015 and June 2016, 24 patients diagnosed with long-standing persistent AF who were scheduled to undergo hybrid ablation surgery were enrolled into the study. Twenty-six patients with sinus rhythm and scheduled for CABG surgery were also enrolled as the non-AF group. We also recruited 25 healthy controls. Baseline demographic and clinical characteristics were collected for all of the patients. Data relating to the duration of AF, history of stroke, and other comorbidities were also gathered. Prior to the surgery, all of the patients underwent transthoracic echocardiographic examination and pulmonary function testing to assess cardiopulmonary function. Enhanced computed tomography of left atrial and pulmonary vein and transesophageal echocardiography were routinely examined to clarify local anatomy and rule out thrombus in the LAA. Autoimmune or infectious diseases were excluded. This study complied with the Declaration of Helsinki and was approved by the Ethics Committee of Beijing Chao-yang Hospital, Beijing, China (13-S-84). All of the subjects were provided a written informed consent before study.

### 2.2. ELISA

Peripheral venous blood samples were acquired from the median cubital vein of the AF patients before hybrid ablation surgery and centrifuged at 1,000*g* for 10 min. Levels of serum anti-M2-R were measured using the ‘‘human muscarinic acetylcholine receptor M2 (mAChRM2) autoantibody enzyme-linked immunosorbent assay (ELISA) kit” (JIANGLAI, Shanghai, People's Republic of China, Catalogue No. JL45683) following the manufacturer's instructions. The variation coefficient between two wells was <5%, and the *R*^2^ values were always >0.98.

### 2.3. Histological Assessment

During surgery, about 200 mg of the LAA were collected. The sample was divided into two parts; one part was rapidly placed into liquid nitrogen and then transferred to a −80°C freezer, and the other was immediately fixed with 10% neutral buffered formalin and then embedded in paraffin. Hematoxylin and eosin (H&E) staining were performed to assess the morphology of myocardial tissue. Evidence of collagen deposition in the extracellular matrix was detected by Masson's trichrome staining. Light microscopy was used to capture high-magnification light micrographs. The image quantitative digital analysis system (Image-Pro Plus 6.0, Media Cybernetics, Inc., Rockville, MD, USA) was used to measure collagen volume fraction (CVF).

### 2.4. Immunohistochemical Analysis

Immunohistochemical staining was performed with paraffin section using the EnVision™ two-step method. M2 receptor antibody (ab2805, Abcam, Cambridge, USA), TGF-*β*1 antibody (ab92486, Abcam), and CTGF antibody (ab6992, Abcam) were added at dilutions of 1 : 100, 1 : 1,000, and 1 : 400, respectively, followed by incubation for 60 min at room temperature and washing three times with phosphate-buffered saline (PBS) solution. Then, 50 *μ*L of EnVision™ reagent was added, and the sections were incubated for 60 min at room temperature and then washed with PBS solution. Finally, the sections were stained with 3,3′-diaminobenzidine and counterstained with hematoxylin. The appearance of brownish yellow granules in the cytomembrane or extracellular matrix demonstrates a positive result.

### 2.5. Immunofluorescence Staining

Indirect immunofluorescence staining was performed to detect the expression of collagen I and collagen III in the paraffin sections of LAA tissues. Tissue sections were blocked with normal bovine serum albumin (Zhongshan Goldenbridge Biotechnology, China) for 30 min, and then, the sections were incubated with polyclonal rabbit anti-human collagen I antibody (1 : 50, ab34710, Abcam) or monoclonal mouse anti-human collagen III antibody (1 : 200, ab6310, Abcam) overnight at 4°C. After washing with PBS solution with 0.1% polysorbate, the sections were incubated with the corresponding secondary antibodies at 37°C for 1 h. Fluorescein isothiocyanate-conjugated goat anti-rabbit IgG (1 : 100, Zhongshan Goldenbridge Biotechnology) was used as the secondary antibody for collagen I, and fluorescein isothiocyanate-conjugated goat anti-mouse IgG (1 : 100, Zhongshan Goldenbridge Biotechnology) was used as the secondary antibody for collagen III. Finally, the sections were washed with PBS solution thrice and counterstained with 4′,6-diamidino-2-phenylindole (1 : 100) for 1 h at room temperature. Negative controls were conducted without incubation with primary antibodies.

### 2.6. Western Blotting

About 50 *μ*g of protein was extracted from the LAA tissues that were stored in the −80°C freezer by lysing in a radio-immunoprecipitation assay lysis buffer. Protein concentration was detected using the bicinchoninic acid assay. An equivalent amount of protein was added to 10% sodium dodecyl sulfate-polyacrylamide gel electrophoresis, and the proteins were transferred onto nitrocellulose membrane. Membranes were blocked with 5% skimmed milk in TBST (10 mM Tris-HCl (pH 8), 150 mM NaCl, and 0.1% Tween 20) for 60 min at room temperature. The primary antibodies were added following the manufacturer's instructions. The nitrocellulose membranes were incubated overnight at 4°C with monoclonal mouse anti-human M2 receptor antibody (1 : 1000, ab2805, Abcam), polyclonal rabbit anti-human TGF-*β*1 antibody (1 : 1,000, ab92486, Abcam), polyclonal rabbit anti-human CTGF antibody (1 : 1,000, ab6992, Abcam), polyclonal rabbit anti-human collagen I antibody (1 : 500, ab34710, Abcam), or monoclonal mouse anti-human collagen III antibody (1 : 500, ab6310, Abcam). Horseradish peroxidase-labeled goat anti-rabbit antibody and goat anti-mouse antibody (1 : 10,000, Zhongshan Goldenbridge Biotechnology) were used as secondary antibodies. After incubation with the secondary antibody diluted in 5% skimmed milk in TBST at room temperature for 40 min, the membranes were washed thrice with TBST, and the film was placed in 10 mL of Ponceau solution for 3 min. The images were placed into an automatic image analyzer, and the density of each band was quantified using the Gel Image System ver.4.00 (Tanon, China). Monoclonal anti-GAPDH antibody was used as the loading control.

### 2.7. Postprocedural Follow-Up

Hybrid ablation surgery was performed according to Pison et al. [14]. All of the patients needed endocardial touch-up ablation to reach bidirectional block. The LAA was removed using a stapling device. All of the patients were transferred to the intensive care unit after hybrid ablation surgery. Amiodarone and warfarin/dabigatran etexilate capsules (Pradaxa®, Boehringer Ingelheim Pharma GmbH and Co., KG) were restarted 6 h after the surgery for three months. If with warfarin, enoxaparin 1 mg/kg was used until the target international normalized ratio of 2.0-3.0 was achieved. Outcomes of hybrid ablation surgery were assessed by off antiarrhythmic drug. All 24 patients were scheduled for visits at 1, 3, 6, and 12 months after hospital discharge and every 6 months thereafter in the outpatient clinics. At each visit, all of the patients received cardiological physical examination, questionnaire for AF-related symptoms (palpitations, fatigue, dizziness, and chest discomfort) and a 12-lead electrocardiogram. Long-attached ambulatory electrocardiographic recorder and software (NS-SP-A-01, Ensense Biomedical Technologies (Shanghai) Co., Ltd.) for 14 days after routine 24 h Holter recording was conducted every 6 months after the hybrid ablation surgery for all of the patients. Any episode of AF, atrial flutter, or atrial tachycardia lasting at least 30 sec and occurring after the 90-day blanking period was classified as recurrence [15].

### 2.8. Statistical Analysis

Quantitative data with normal distribution were presented as the mean ± standard deviation. Qualitative data were presented as frequencies and percentages. Quantitative data were compared by one-way ANOVA and unpaired Student's *t*-test among the three groups or between the two groups. Qualitative data were compared using the chi-square test. Pearson correlation analysis was used to test the correlation between serum anti-M2-R levels and fibrosis of LAA-related parameters. Statistical analysis was performed using SPSS statistical software (version 20.0; SPSS Inc.). A two-tailed *p* < 0.05 was considered to be statistically significant.

## 3. Results

### 3.1. Patients Characteristics and Clinical Findings

The patients' basic information and echocardiographic data are shown in Table 1. Demographic characteristics were similar among the three groups. Comorbidities were similar between the AF and the non-AF groups. There were no significant differences in pulmonary function and echocardiographic data, including left ventricular end-diastolic dimension and left ventricular ejection fraction among the three groups. The mean left atrium diameter of the AF group estimated by echocardiography was significantly larger than that in the non-AF group (58.0 ± 5.3 mm vs. 36.3 ± 1.1 mm; *p* < 0.001).

In the AF group, the duration of AF was 103.3 ± 61.8 months. The CHA2DS2-VASc score was 2.6 ± 1.8, and HAS-BLED score was 1.3 ± 1.0. The pulmonary function test and the anatomy of left atrium and pulmonary vein were normal. All 24 of the patients underwent hybrid ablation surgery and LAA excision successfully. The mean anesthesia duration was 375.6 ± 118.6 min, and the mean duration of operation was 280.9 ± 117.3 min. The hospital stay was 20.1 ± 8.4 days. At the two-year follow-up, the success rate of hybrid ablation was 87.5% (21/24).

### 3.2. Elevated Serum Anti-M2-R and M2 Receptor in AF Patients

**Figure 1:**
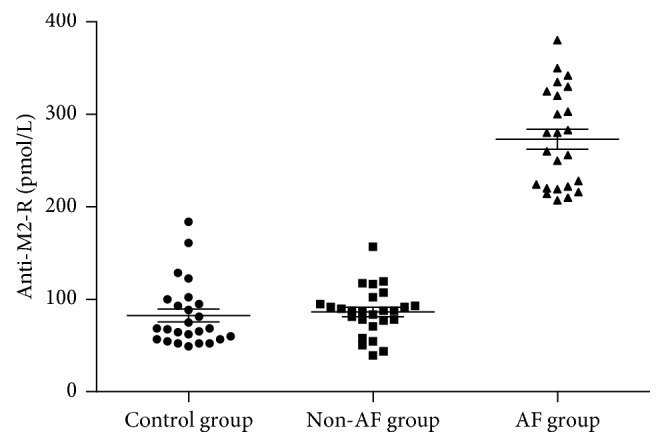
Comparison in serum anti-M2-R levels among three groups. The serum anti-M2-R level in the AF group was 496.2 ± 232.5 pmol/L, which was significantly higher than 86.3 ± 25.7 pmol/L in the non-AF group, *p* < 0.001. No difference in serum anti-M2-R levels between the non-AF and control group was observed (86.3 ± 25.7 vs. 82.4 ± 34.9 pmol/L, *p*=0.654).

**Figure 2:**
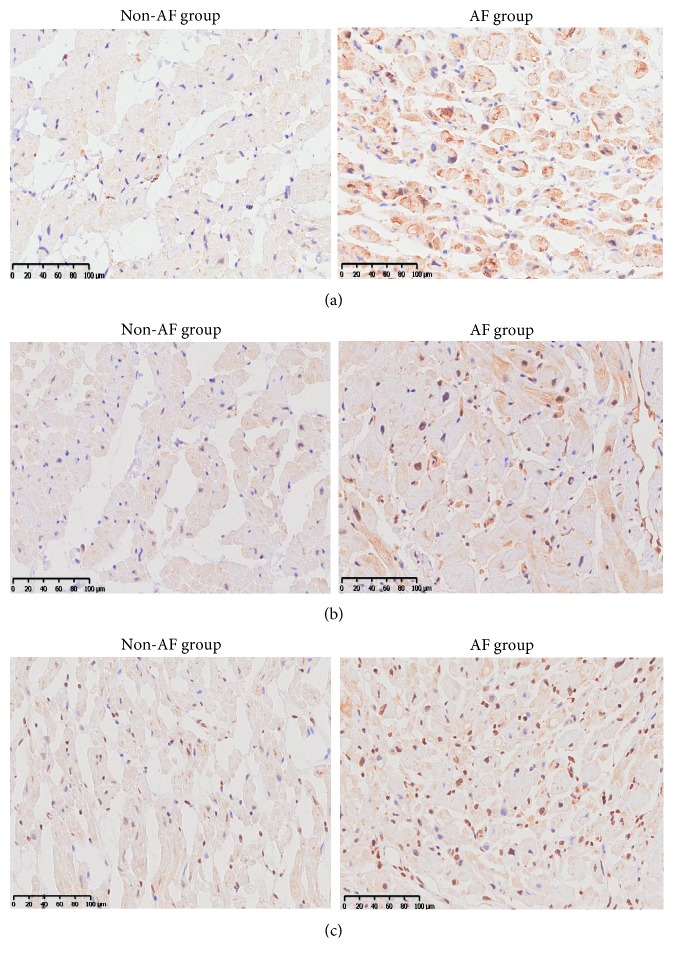
Immunohistochemical analysis of M2 receptor, TGF-*β*1, and CTGF expression. Representative sections of the immunohistochemistry- (IHC-) stained left atrial appendage tissue of patients in the non-AF and AF group. (a) M2 receptor; (b) TGF-*β*1; (c) CTGF.

**Figure 3:**
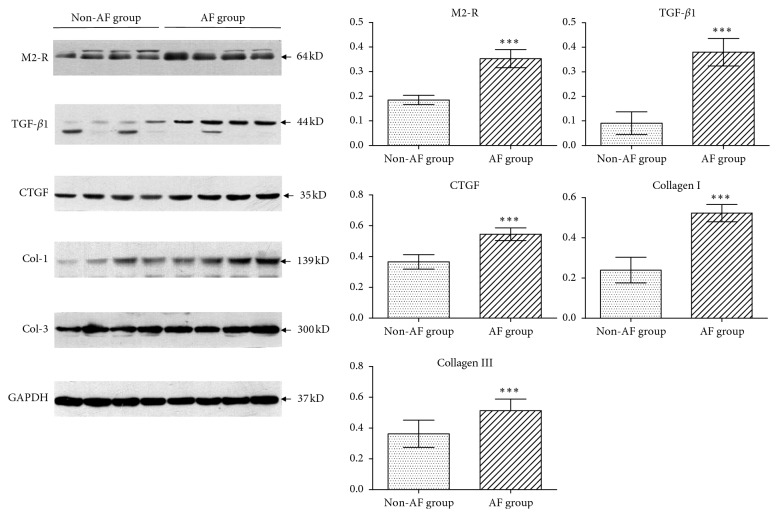
Western blotting analysis. Western blotting examined the left atrial appendage of patients in the non-AF group and the AF group. Left, representative western blot analysis the expression in the non-AF group and the AF group; right, quantitative results of the protein levels. ^*∗∗∗*^*p* < 0.001. GAPDH, glyceraldehyde-3-phosphate dehydrogenase.

**Figure 4:**
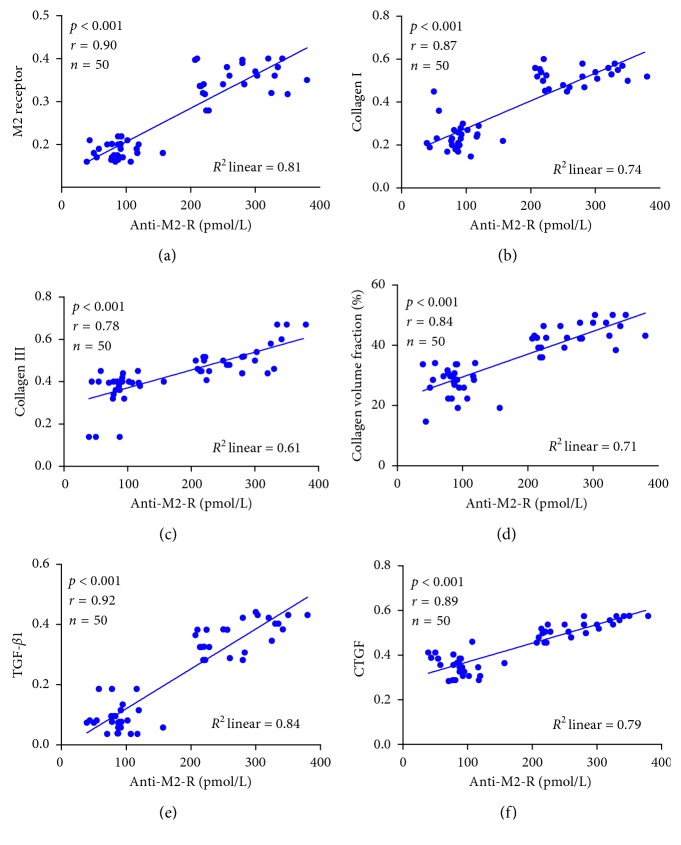
Correlation between serum anti-M2-R levels and fibrosis-related indexes. Pearson correlation analysis showed that M2 receptor, CVF, and collagen I and collagen III were all correlated with serum anti-M2-R levels (M2 receptor: *r* = 0.90, *p* < 0.001; CVF: *r* = 0.84, *p* < 0.001; collagen I: *r* = 0.87, *p* < 0.001; collagen III: *r* = 0.78, *p* < 0.001). Fibrogenic indexes were also correlated with serum anti-M2-R levels (TGF-*β*1: *r* = 0.92, *p* < 0.001; CTGF: *r* = 0.89, *p* < 0.001).

Serum anti-M2-R levels were significantly higher in the AF group compared to the non-AF group (496.2 ± 232.5 vs. 86.3 ± 25.7 pmol/L, *p* < 0.001). There were no differences between the non-AF and the control groups (86.3 ± 25.7 vs. 82.4 ± 34.9 pmol/L, *p*=0.654) (Figure 1). The expression of M2 receptor was determined by immnunohistochemical and Western blotting analyses (Figures 2(a) and 3). M2 receptor expression was higher in the AF group than the non-AF group (0.35 ± 0.04 vs. 0.18 ± 0.02, *p* < 0.001). Pearson correlation analysis showed a close relationship between serum anti-M2-R and M2 receptor levels (*r* = 0.90, *p* < 0.001), Figure 4.

### 3.3. Occurrence of Atrial Fibrosis in AF Patients

**Figure 5:**
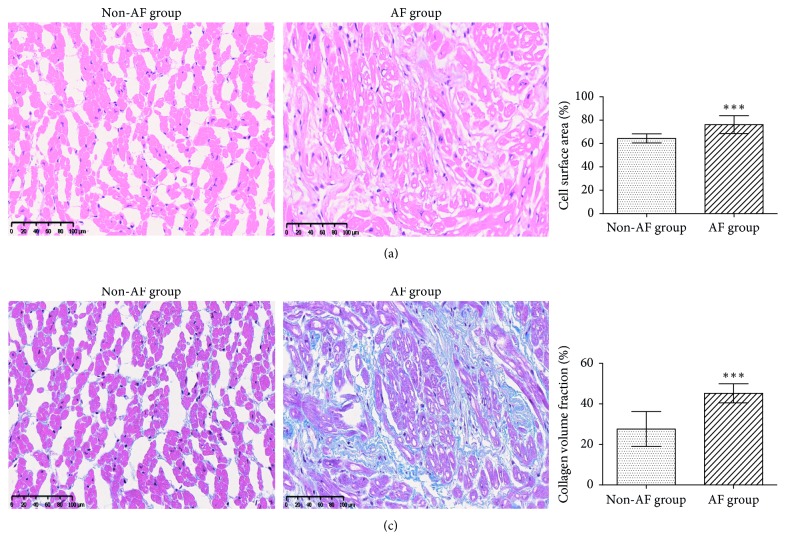
Histological analysis of the left atrial appendage. (a) Hematoxylin and eosin (H&E) staining of the left atrium appendage in the non-AF and the AF groups. Left, representative image; right, statistical results for the cell surface area. (b) Masson's trichrome staining of the left atrium appendage in the non-AF and AF groups. Left, representative image; right, quantification of the collagen volume fraction. ^*∗∗*^*p* < 0.01; ^*∗∗∗*^*p* < 0.001.

Representative sections of LAA tissues from the two groups subjected to H&E and Masson's trichrome staining are shown in Figure 5. H&E staining of sections from the AF group indicated hypertrophy, vacuolar degeneration, and necrosis of cardiomyocytes. The cell surface area increased in the AF group compared to the non-AF group (76.2 ± 7.7% vs. 64.4 ± 3.9%, *p* < 0.001; Figure 5(a)). There were abundant collagen fibers distributed in the extracellular matrix in the AF group. CVF increased in the AF group than in the non-AF group (45.2 ± 4.7% vs. 27.6 ± 8.3%, *p* < 0.001; Figure 5(b)).

**Figure 6:**
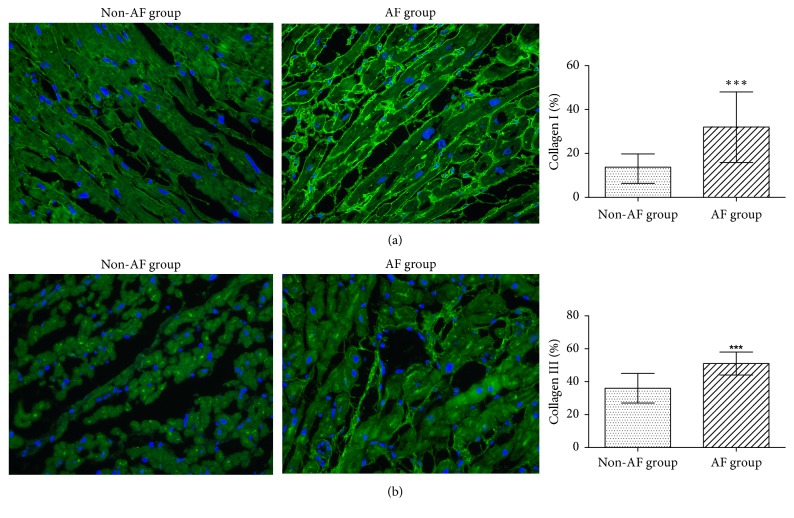
Immunofluorescence analysis the expression of collagen I and collagen III. Immunofluorescence staining of the left atrial appendage of patients in the two groups. Left, representative image; right, statistical results for the collagen (a) I and (b) III. ^*∗∗∗*^*p* < 0.001; magnification, 400×.

Furthermore, we determined the expression of collagen I and collagen III by immunofluorescence staining and Western blotting (Figures 3 and 6). Although collagen I and collagen III were expressed in both groups, the levels were considerably higher in the LAA of the AF group than in the non-AF group (collagen I: 0.52 ± 0.04 vs. 0.24 ± 0.06, *p* < 0.001; collagen III: 0.51 ± 0.07 vs. 0.36 ± 0.09, *p* < 0.001).

### 3.4. Increased Fibrogenic Indexes in AF Patients

The expression of TGF-*β*1 and CTGF were determined by immnunohistochemistry and Western blotting (Figures 2(b)-2(c) and 3). These indicators all increased in the AF group compared to the non-AF group (TGF-*β*1: 0.38 ± 0.06 vs. 0.09 ± 0.04, *p* < 0.001; CTGF: 0.55 ± 0.04 vs. 0.37 ± 0.05, *p* < 0.001).

### 3.5. Correlation between Fibrosis and Serum Anti-M2-R Levels

CVF and collagen I and collagen III were all correlated with serum anti-M2-R levels (CVF: *r* = 0.84, *p* < 0.001; collagen I: *r* = 0.87, *p* < 0.001; collagen III: *r* = 0.78, *p* < 0.001). Fibrogenic indexes were also correlated with serum anti-M2-R levels (TGF-*β*1: *r* = 0.92, *p* < 0.001; CTGF: *r* = 0.89, *p* < 0.001), Figure 4.

## 4. Discussion

### 4.1. Major Findings

Our findings indicate that serum anti-M2-R levels are significantly higher in AF patients and are correlated with the severity of atrial fibrosis as shown by histology and molecular biology assays. Atrial fibrosis is probably one of the important factors between anti-M2-R and the occurrence and maintenance of AF. Our results further revealed a positive correlation between serum anti-M2-R levels and TGF-*β*1 and CTGF expression in LAA tissues. These findings suggest that serum anti-M2-R plays an important role in fibrosis-associated AF and its potential mechanism.

### 4.2. Elevated Serum Anti-M2-R Levels in AF Patients

Anti-M2-R was first detected in idiopathic dilated cardiomyopathy patients in 1993 by Fu et al. [16]. Later, anti-M2-R was reported as the strongest independent predictor for the presence of AF in patients with idiopathic dilated cardiomyopathy and Graves' hyperthyroidism [17]. We further found that the frequency of anti-M2-R in AF patients with normal heart function was 40.8%. Yalcin et al. found that the serum anti-M2-R level was 142.3 ng/mL in lone paroxysmal AF patients, markedly higher than 69.0 ng/mL in healthy controls. Using a cutoff value of 101.83 ng/mL for predicting the presence of lone paroxysmal AF, the sensitivity was 94.68% and the specificity was 81.33% [18]. In this study, we found that the serum anti-M2-R level in long-term persistent AF patients was 496.2 pmol/L, significantly higher than 86.3 pmol/L in non-AF controls. Some investigators have suggested that anti-M2-R probably is a novel mediator or upstream target in AF [6, 19, 20].

### 4.3. Role of Serum Anti-M2-R in the Pathogenesis of AF

The atrial electrical, structural, and autonomic remodeling has been recognized to contribute to the maintenance of AF. Circulating anti-M2-R can recognize and bind to 169–193 amino acids of the second extracellular loop of the M2 receptor specifically. It is not only able to bind to the target receptor in the myocardium but also to induce biological responses as partial agonist [16, 21]. Anti-M2-R binds to the M2 receptor, resulting in the activation of the muscarinic-gated potassium channel *I*_K,Ach_, and the primary cellular electrophysiological effects are hyperpolarization and shortening of action potential duration [22]. In knockout mice lacking this channel, M-receptor stimulation did not induce AF [23]. Thereafter, Hong et al. [22] reported that atrial tissues clearly exhibit atrial fibrosis in rabbits immunized with a synthetic peptide corresponding to the M2 receptor. DE-MRI has recently been used for visualizing left atrial fibrosis for AF ablation procedures to estimate the degree of atrial fibrosis. Gurses et al. [9] showed that serum anti-M2-R levels may be associated with the severity of left atrial fibrosis as quantified by DE-MRI. However, the histological changes and underlying molecular mechanism have not been extensively studied. In this study, we quantified serum anti-M2-R levels and we found that they are associated with CVF and fibrogenic indexes related to fibrosis. These results agree with the findings of previous studies on the ventricles [24, 25]. Earlier investigations have demonstrated that the mRNA level of the inhibitory G protein *α* subunit is higher in heart failure patients and pace-induced heart failure dogs [26, 27]. Redfern et al. [28] reported that fibronectin, laminin, and collagen were all upregulated in transgenic mice that conditionally expressed receptors that constitutively activated the inhibitory G protein signaling pathway. We hypothesize that the cause of atrial fibrosis in these AF patients may be via the activation of the inhibitory G protein pathway.

### 4.4. Increased M2 Receptor Expression in AF Patients

Previous studies have shown that AF dogs with vagal stimulation can significantly increase the expression of M2 receptor in the atrium, particularly in the atrial appendage, which indicated that the atrial appendage perhaps play an important role in initiation of cholinergic AF [29]. The density of M2 receptor in the left atrium increases with aging in rabbits, which contributed to the increased age-related AF vulnerability [30]. Hong et al. [22] found the expression of the M2 receptor in the atrium of anti-M2-R positive rabbits was significantly upregulated. Our study found that the expression of M2 receptor is higher in AF patients compared to the non-AF group, which agrees with the results of previous reports on rabbits [22, 30]. Zhao et al. [31] proposed that the remodeling of the M2 receptor might not be associated with AF, but with the dilated left atrium. Cisatracurium, a neuromuscular blocker, with allosteric binding to the M2 receptor in the atrium, demonstrates a dose-dependent suppression of AF and shortening of the atrial action potential accompanied by vagus nerve stimulation without facilitating sinus or atrioventricular nodal function [32]. Selective M2 receptor antagonist may thus be potentially utilized as a novel therapeutic target for the treatment of AF patients.

### 4.5. TGF-*β*1 and CTGF in Atrial Fibrosis

Data from biopsy specimens of patients with AF have uncovered the presence of atrial fibrosis [33]. Collagen I and collagen III, the major matrix proteins of cardiomyocytes, constitute 85% of the matrix. TGF-*β*1 is a key regulator of fibrosis that enhances collagen synthesis [11, 34]. CTGF is upregulated via the TGF-*β*1/Smad pathway in the atrial myocardium of AF patients [12]. In this study, we found that the expression of TGF-*β*1 and CTGF in LAA tissues is correlated with serum anti-M2-R levels. It is reported that muscarinic receptors exert stimulatory effects on collagen synthesis in human lung fibroblasts [35], and crosstalk between TGF-*β*1 and M2 receptor augments airway smooth muscle proliferation [36]. Earlier studies have shown that TGF-*β*1 stimulation of chick heart cells results in the downregulation of the M2 receptor and muscarinic responsiveness [37], while we observed the upregulation of M2 receptor and TGF-*β*1 expression in LAA in AF patients. As the small number of patients enrolled in this study, further study is needed to verify the association between M2 receptor and TGF-*β*1.

### 4.6. Limitations

This study has a number of limitations. First, this is a single-center, small-sample population study. The results may thus be exaggerated for selection bias, and they cannot be directly expanded to all AF patients. A multicenter, large-sample population study is thus needed to verify our results. Second, as none of the patients had an implanted internal loop recorder, AF recurrence might be underestimated. Finally, this is a clinical study on the association between serum anti-M2-R levels and fibrosis of LAA. These results do not reveal a causal relationship but only demonstrate a correlation. The molecular mechanism of anti-M2-R in atrial fibrosis in AF patients require further investigation.

## 5. Conclusions

Serum anti-M2-R levels are significantly higher in AF patients and are associated with the severity of atrial fibrosis. In addition, a positive correlation between serum anti-M2-R levels and the expressions of TGF-*β*1 and CTGF in LAA tissues in AF patients was observed.

## Figures and Tables

**Table 1:** Baseline characteristics of the control group and study population.

	Control group (*n* = 25)	AF group (*n* = 24)	Non-AF group (*n* = 26)
*Clinical parameters*			
Age (years)	65.6 ± 7.8	62.5 ± 9.9	65.8 ± 7.3
Gender, male (%)	13 (52.0)	16 (66.7)	14 (53.8)
Smoking (%)	9 (36.0)	9 (37.5)	10 (38.5)
Alcohol consumption (%)	7 (28.0)	8 (33.3)	8 (30.8)
BMI (kg/m^2^)	25.4 ± 2.9	26.6 ± 3.8	25.7 ± 3.1

*Comorbidity*			
Hypertension (%)	0	12 (50.0)	14 (53.8)
Diabetes mellitus (%)	0	9 (37.5)	10 (38.5)
History of stroke (%)	0	10 (41.7)	11 (42.3)

*Echocardiographic parameters*			
Left atrium diameter (mm)	36.0 ± 1.2	58.0 ± 5.3^*∗*^	36.3 ± 1.1
LVEDD (mm)	48.9 ± 1.8	50.0 ± 3.3	49.2 ± 2.0
LVEF (%)	62.4 ± 4.3	60.3 ± 8.6	62.1 ± 5.0

*Pulmonary function*			
FEV1 (L)	2.8 ± 0.5	2.6 ± 0.4	2.6 ± 0.2
FEV1/FVC (%)	77.6 ± 2.8	76.6 ± 4.1	76.8 ± 3.6

BMI, body mass index; LVEDD, left ventricular end diastolic diameter; LVEF, left ventricular ejection fraction; Anti-M2-R, autoantibodies against M2-muscarinic acetylcholine receptors. One-way ANOVA and the unpaired Student's two-tailed *t*-test were performed among the three groups or between two groups. ^*∗*^*p* < 0.001.

## Data Availability

The data used to support the findings of this study are available from the corresponding author upon request.
